# Clinical Imaging Findings of Vestibular Aqueduct Trauma in a Patient With Posttraumatic Meniere's Syndrome

**DOI:** 10.3389/fneur.2019.00431

**Published:** 2019-04-25

**Authors:** David Bächinger, Madeline M. Goosmann, Bernhard Schuknecht, Joseph B. Nadol, Joe C. Adams, Alexander Huber, Andreas H. Eckhard

**Affiliations:** ^1^Department of Otorhinolaryngology, Head and Neck Surgery, University Hospital Zurich, Zurich, Switzerland; ^2^University of Zurich, Zurich, Switzerland; ^3^Medical Radiological Institute MRI, Zurich, Switzerland; ^4^Department of Otolaryngology, Harvard Medical School, Boston, MA, United States; ^5^Otopathology Laboratory, Massachusetts Eye and Ear Infirmary, Boston, MA, United States

**Keywords:** endolymphatic sac, endolymphatic hydrops, temporal bone fracture, otic capsule, gadolinium-enhanced magnetic resonance imaging

## Abstract

Posttraumatic Meniere's syndrome is a rare clinical entity. The pathomechanism by which temporal bone trauma leads to fluctuating audiovestibular symptoms, in some cases with a delay of onset many years after trauma, remains elusive. Here, a clinical case and the respective temporal bone imaging data were reviewed to investigate the underlying inner ear pathology. A 44-year-old patient presented with left-sided Meniere's syndrome 34 years after he suffered an ipsilateral temporal bone fracture caused by a car accident. Clinical imaging showed left cochleovestibular hydrops (gadolinium-enhanced MRI) and bony obliteration of the left VA (CT imaging), resulting in discontinuity of the ES. Our findings suggest that a temporal bone fracture with a “retrolabyrinthine” course, traversing the VA, caused intraaqueductal callus bone formation and progressive blockage of the VA. As a result, the extraosseous (distal) endolymphatic sac (eES) became separated from the cochleovestibular labyrinth, an event that presumably underlies endolymphatic hydrops formation and that precipitates the onset of clinical Meniere's symptoms in this case.

## Background

Meniere's disease (MD) is an idiopathic inner ear disorder that presents with episodic vertigo, fluctuating hearing loss, tinnitus, and aural fullness (1, 2). In addition to an idiopathic etiology, certain identifiable predisposing causes, including temporal bone (TB) fractures, can be associated with Meniere's symptoms [reviewed in Merchant and Nadol (3)] which are then referred to as “Meniere's syndrome” (4). In the literature, only a few postmortem histopathology cases with TB fracture-associated (posttraumatic) Meniere's syndrome are documented (5–7). Although fracture lines through the vestibular aqueduct (VA) were a common finding among those pathology cases, the pathomechanism that leads to the onset of posttraumatic Meniere's syndrome, sometimes years after the injury, is not understood. Moreover, radiological criteria to identify TB fractures that will cause posttraumatic Meniere's syndrome are lacking.In a recent histopathology study on a large series of MD cases, we consistently found pathological changes in the extraosseous portion of the endolymphatic sac (eES) (8). Because the normal human eES epithelium carries out several distinct ion transport functions, which are believed to be crucial for endolymph homeostasis, we consider those eES pathologies to be of critical significance in the etiopathogenesis of endolymphatic hydrops, as well as for the clinical symptomatology in MD (8).Herein, we retrospectively investigated imaging data from a clinical case of posttraumatic Meniere's syndrome for signs of traumatic eES pathology, based on the hypothesis that TB trauma may have led to the same inner ear pathophysiology—i.e., loss of eES function—that was previously observed in MD.

## Case Presentation

### Clinical History

A 44-year-old man was referred to our interdisciplinary center for vertigo and balance disorders at the University Hospital Zurich (tertiary referral center) with recurrent spontaneous attacks of spinning vertigo, which started several months prior to his first visit. The attacks usually lasted for 3–12 h and were accompanied by fluctuating hearing loss and tinnitus in the right ear.

**Figure 1:**
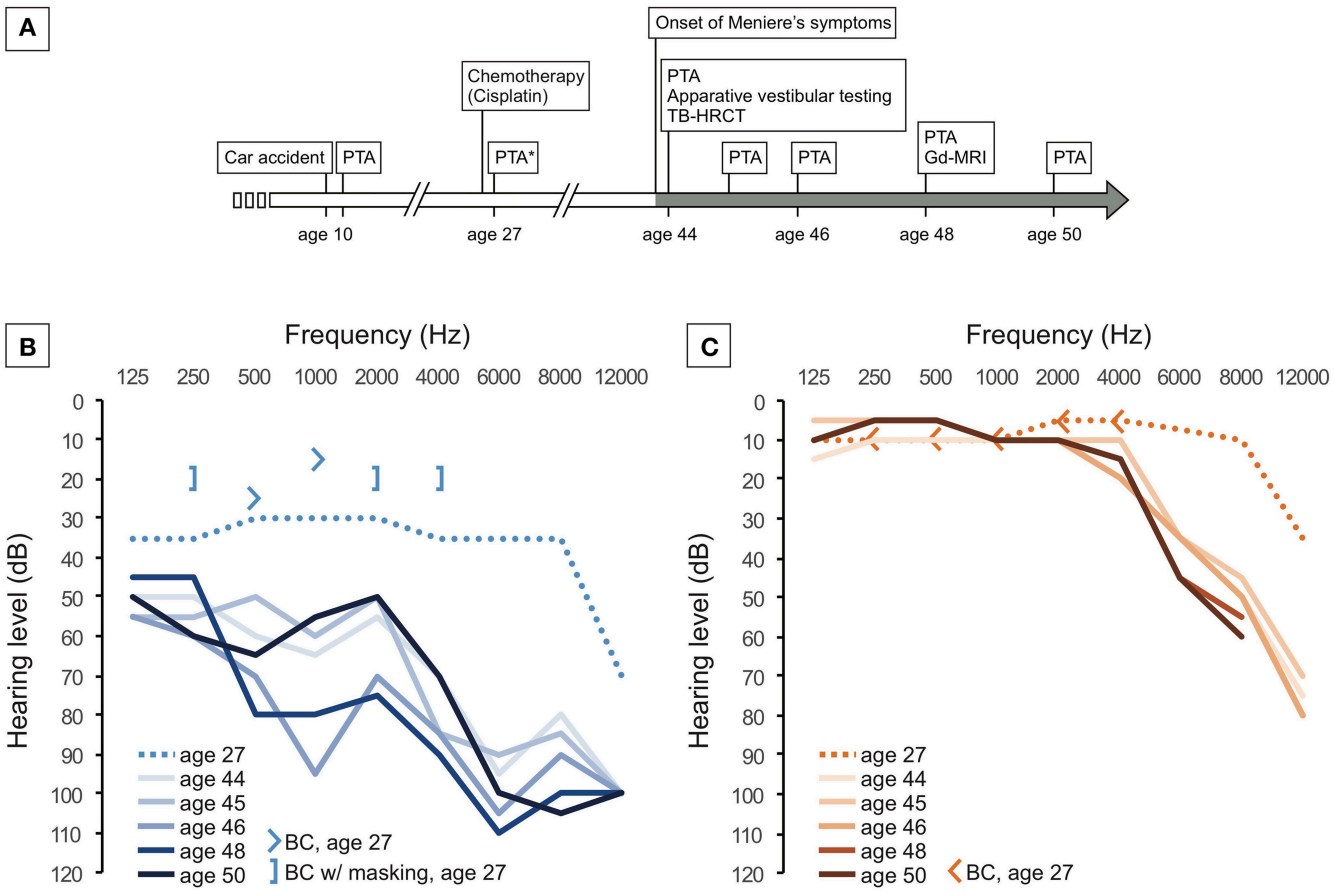
**(A)** Timeline of the patient's medical history and of diagnostic interventions. **(B,C)** Repeated pure-tone audiometric recordings for the left ear **(B)** and right ear **(C)** between age 27 and 50 years. Curves indicate air conduction thresholds. Arrows and brackets indicate bone conduction (BC) thresholds, as measured at age 27.

According to the patient's medical history, he had suffered a left-sided longitudinal TB fracture caused by a car accident at 10 years of age (the original neuroradiology report but not the CT images were available for this study). Pure-tone audiometry (PTA) 4 months after the accident showed a pronounced high-frequency shift in bone conduction thresholds at 6 kHz on the left side (PTA not shown here), consistent with acoustic trauma and probably caused by the impact noise in the car. No further accident-related injuries or audiovestibular symptoms occurred, according to the available clinical records from that time. A synopsis of relevant events in the patient's medical history is given in Figure 1A.

### Clinical Presentation, Diagnosis, and Disease Course

In the initial neurotological work-up at age 44, vestibular-evoked myogenic potentials (VEMPs) indicated left-sided saccular dysfunction (absent cervical VEMPs). Other vestibular test results (ocular VEMPs, subjective visual vertical, video-oculography with caloric stimulation, and video head impulse test) were within the normal range (data not shown). PTA showed that the left ear had mixed, predominantly sensorineural, downward-sloping hearing loss (HL) up to 100 dB HL at 6 kHz (Figure 1B), while the right ear had moderate to severe presbyacusis (Figure 1C). Speech discrimination scores were 35 % on the left side and 100 % on the right side, and stapedial reflex responses were normal on both sides (data not shown).

Two months later, the patient presented in our emergency department immediately after an acute, hours-long vertigo attack accompanied by nausea and emesis. The HL and tinnitus in the left ear temporally worsened. Clinical examination showed a spontaneous nystagmus to the right side, a positive head impulse test to the left, and a positive Romberg test with a tendency to fall to the left side. Finally, a diagnosis of left-sided Meniere's syndrome (1, 2) was made, and a possible traumatic etiology was considered based on the patient's history.

The patient subsequently received repeated intratympanic dexamethasone (6.6 mg)/hyaluronate (2 mg/ml) injections over the course of several days, and in the following years, he undertook sequential trials of different vestibular suppressants [cinnageron, 75 mg, two times daily for 8 weeks, betahistine, 24 mg, two times daily for 16 weeks; prior to publication of (9)]. Although the vertigo episodes continued with an average frequency of two attacks per month, the patient was coping well with the symptoms, and no vestibular ablative treatment was considered. In the 4 years following the onset of MD symptoms, hearing function in the left ear fluctuated in the low and middle frequencies and progressively declined to a level of 50–80 dB HL (Figure 1B). Finally, the patient was fitted with a contralateral routing of signals (CROS) hearing aid. He was then lost to further follow-up.

### Clinical Imaging

During initial neurotological evaluation (age 44), high-resolution CT (HRCT) imaging of the TBs was performed to rule out retrocochlear pathologies (e.g., vestibular schwannoma), posttraumatic inclusion cholesteatoma, and otosclerosis. HRCT imaging showed no evidence for the previously mentioned pathologies but demonstrated an old fracture sign in the posterior wall of the external ear canal. Four years later (age 48), gadolinium-enhanced MRI (Gd-MRI) of the inner ear fluid spaces with a 4-h delayed 3D inversion recovery (3D IR) sequence (10, 11) detected moderate [grade I, (11)] vestibular and moderate-to-severe [grade I-II, (11)] cochlear hydrops in the left inner ear, consistent with the previously assigned clinical diagnosis. No radiological (Gd-MRI) signs of endolymphatic hydrops were found in the right inner ear.

**Figure 2:**
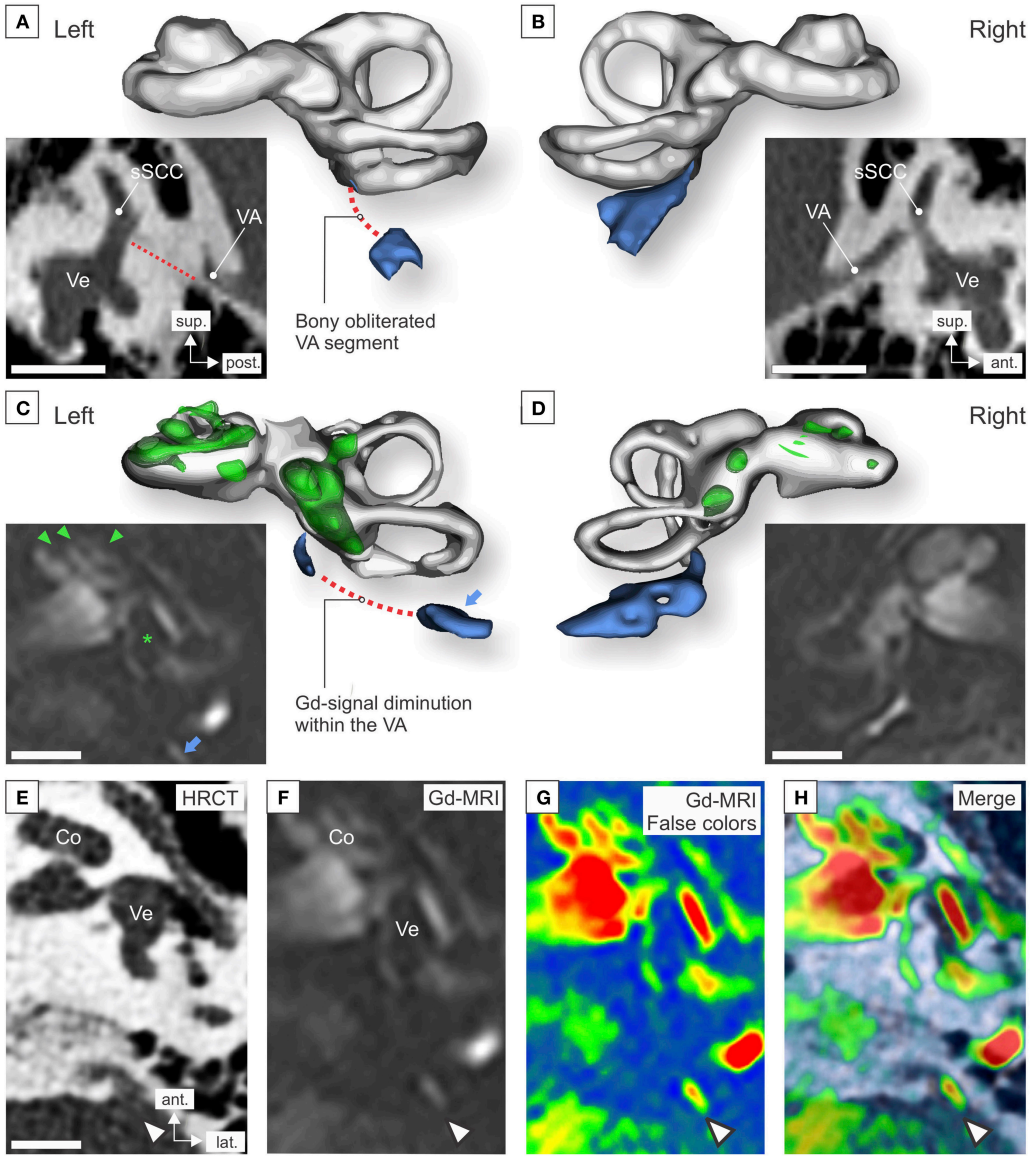
High-resolution CT (HRCT) imaging and gadolinium-enhanced MRI (Gd-MRI) of the inner ears was performed after the onset of Meniere's symptoms (HRCT, at age 44; Gd-MRI, at age 48). **(A,B)** Sagittal HRCT projections and 3D segmentations of the left **(A)** and right **(B)** labyrinth. (VA, vestibular aqueduct (blue in 3D segmentation); sSCC, superior semicircular canal; Ve, vestibule; red dotted line in **(A)**, bony obliterated VA. **(C,D)** Axial Gd-MRI (3D inversion recovery sequence) and 3D segmentations of the left **(C)** and right **(D)** labyrinth. Signal voids in the cochlear turns (green arrowheads) and the saccule (green asterisk) indicate endolymphatic hydrops; blue arrow, signal originating from the extraosseous endolymphatic sac. 3D segmentations were performed using the 3D Slicer software [version 4.6.2, (12)]. **(E–H)** Fusion of axial HRCT **(E)** and Gd-MRI [**F**; false color-coded image in **(G)**] images of the left temporal bone in the corresponding axial plane **(H)**. Arrowheads **(E–H)**: T2 signal localized posterior to the operculum, corresponding to the localization of the distal (extraosseous) endolymphatic sac **(D)**. (Co, cochlea; PSCC, posterior semicircular canal; VA, vestibular aqueduct; Ve, vestibule). Scale bars: 5 mm. The false color-coding image overlay was generated using Adobe Photoshop CC2017 (version 18.1.0, San Jose, CA, USA).

In the present study, we reassessed the available imaging data with regard to potential pathologies of the VA and ES on the clinically affected left side. On HRCT imaging, the left VA appeared to be almost completely obliterated by bone along its course between the isthmus and the operculum (Figure 2A). In contrast, the right VA exhibited a patent lumen throughout its entire course (Figure 2B). Correspondingly, on Gd-MRI (3D IR sequence), a segment of total T2 signal loss was observed in the partially obliterated left VA (Figure 2C) but not in the right VA (Figure 2D). In the left VA, a delineated T2 signal was seen distal (posterior) to the segment with total signal loss (Figure 2C, blue arrows). Fusion of HRCT and Gd-MRI (color-coded) data in the focal plane of the operculum clearly localized this T2 signal distal (posterior) to the operculum (Figures 2E–H). Therefore, the signal most likely originated from the eES, as proposed previously (13). In summary, the HRCT and Gd-MRI data indicated that the left eES was structurally preserved but anatomically separated from the labyrinth due to bony obliteration of the VA. Upon reassessment of the imaging data for the present study, no radiological signs for bony labyrinthine dehiscence, nor for cerebellopontine angle tumors were found that could account for the fluctuating otological symptoms.

## Discussion

**Figure 3:**
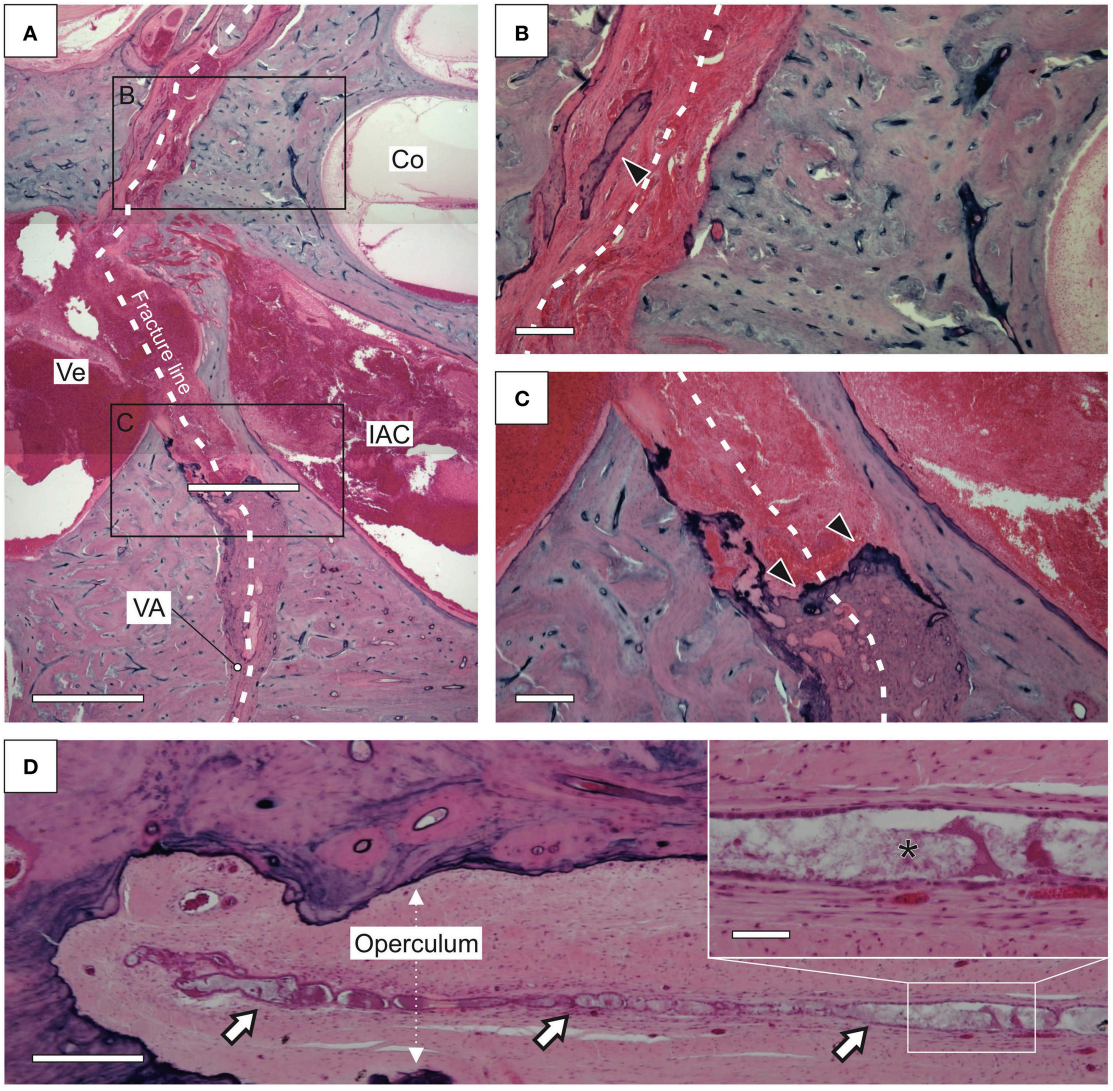
Histopathology of a temporal bone fracture that affected the vestibular aqueduct (VA) in a postmortem case [same as “case 1” in (7)]. The patient died 33 years after the trauma. **(A–C)** Overview **(A)** and details **(B,C)** of the fracture line (white dotted line) that spared the cochlea (Co) and coursed through the vestibule (Ve) into the VA. In close proximity to the labyrinth spaces of the Co and the Ve, the fracture cleft is filled only with fibrous tissue, and only minor bony fracture healing [new bone formation; arrowhead in **(B)**] was observed. In the VA, at an increased distance from the Co and the Ve, the fracture cleft became entirely obliterated with new callus bone [arrows in **(C)**]. **(D)** The endolymphatic sac distal to the operculum (extraosseous portion) demonstrated a normal epithelium (IAC, internal auditory canal). The inner ear presented cochleosaccular endolymphatic hydrops (data not shown). Scale bars: **(A,D)**, 500 μm; (**B,C**, inset in **D**), 100 μm. Courtesy of the Massachusetts Eye and Ear Infirmary (MEEI; Boston, MA, USA).

**Figure 4:**
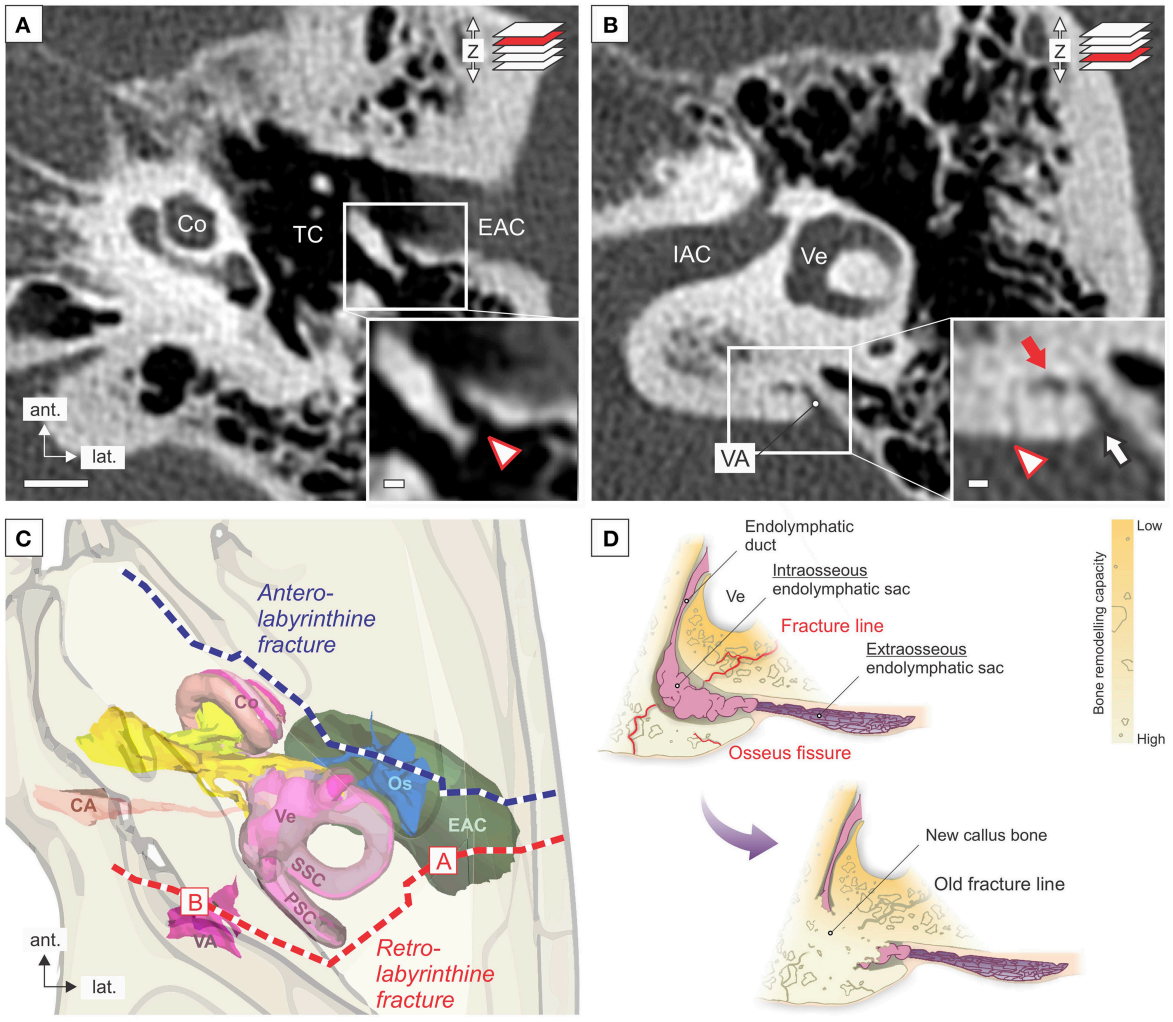
Proposed retrolabyrinthine fracture course in the present case and its impact on the VA. **(A,B)** Old fracture signs (age 44) in the posterior wall of the external auditory canal [EAC; **(A)**] and in the opercular region **(B)** on HRCT imaging. **(C)** Illustration of the trajectory of the proposed fracture in the present case (red line) and the common trajectory of a longitudinal temporal bone fracture (blue line). Localizations of these fracture signs are marked in **(C)**. **(C)** Illustration of the proposed fracture trajectory in the present case (red line), as well as a common longitudinal fracture (blue line). **(D)** Hypothesized mechanism of secondary fracture healing and new callus bone formation with consecutive blockage of the VA and separation of the eES in a fracture that courses through the VA outside the non-remodeling otic capsule bone. Scale bars: **(B)**, 5 mm; (**B**, inlay), 1 mm. The illustration in **(C)** was generated using the 3D viewer of the Human Temporal Bone model [Eaton Peabody Laboratories, MEEI, (18)].

The case described here presented an unusual course for a TB fracture, which spared the cochlea and the vestibular labyrinth but affected the VA in the opercular region. Fractures within the otic capsule, i.e., the bony layer, which encases the cochleovestibular labyrinth, are not repaired by new bone formation (14) due to the lack of capacity for bone growth, modeling, remodeling, and fracture healing in the otic capsule bone (15–17). The VA, however, stretches beyond the otic capsule to the operculum through a layer of bone with normal remodeling and repair capacity [Figure 3; (14)]. A fracture within this retrolabyrinthine region of the petrous bone, as in the present case (Figures 3, 4A–C), would induce normal bone repair mechanisms, including new callus bone formation (Figure 4D). In the present case, as well as in an unrelated postmortem case (Figure 3) with a very similar retrolabyrinthine fracture trajectory and ipsilateral severe cochleosaccular hydrops (hydrops not shown here), the fracture cleft that crossed the VA was healed by excessive new callus bone formation, which adversely obliterated the VA lumen (Figures 2A, 3C). The eES, although not affected by the initial trauma, as indicated by a normal T2-signal in Gd-MRI (Figures 2E–H), was anatomically and functionally separated from the cochleovestibular labyrinth by the blocked VA (Figure 4D). This pathology resembles the surgical endolymphatic duct blockage procedure, which in different animal species leads to the development of endolymphatic hydrops (19). Notably, in a recent human pathology study, loss of the eES was linked to the pathophysiology of endolymphatic hydrops and Meniere's symptoms in (idiopathic) MD (8). In this previous study, two different etiologies of eES pathology were identified among MD patients, i.e., degenerative change in the eES epithelium and developmental hypoplasia of the ES with absence of the eES portion (8, 20). We therefore consider loss of eES function, although of fundamentally different etiologies, to be the common pathophysiological basis of endolymphatic hydrops and Meniere's symptoms in the present case of posttraumatic Meniere's syndrome, as well as in cases of (idiopathic) MD. The molecular pathophysiology that results from the trauma-induced separation of the eES is presumably related to the loss of distinct ion transport mechanisms that are exclusively localized in the eES epithelium (8, 21, 22). With regard to the extensive time interval between TB trauma and the onset of Meniere's symptoms in the present case (34 years) and in many previously reported cases [up to 40 years, (5, 23–25)], it is significant to note that in patients with (idiopathic) MD and associated ES hypoplasia, Meniere's symptoms, on average, do not manifest before the fourth decade of life (8, 26). Thus, loss/absence of eES function—due to trauma or developmental malformation—may be compensated by other epithelial sites in the inner ear over a long period of time. Another factor contributing to the long delay of clinical sequelae in the present case may have been a slowly progressive obliteration of the VA by new callus bone that formed at the fracture site and that did not lead to an immediate but rather a progressive or delayed loss of eES function.

Predicting inner ear sequelae of TB fractures is of particular clinical relevance. The clinicoradiological classification into “otic capsule-sparing (OCS)” vs. “otic capsule-violating (OCV)” fractures (27–30) exhibits the highest predictive value for inner ear sequelae in OCV fractures. This classification, however, does not adequately consider the fracture in the present case, which, by definition, exhibited an OCS trajectory, i.e., the fracture line did not course into or through the cochlea or the labyrinth (vestibule, semicircular canals), although it caused inner ear sequelae. Nevertheless, the fracture did violate a portion of the labyrinth outside the otic capsule bone—the VA that harbors the ES. In conclusion, for the radiological assessment of TB fractures, the entire VA should be regarded as a part of the otic capsule, and delayed inner ear sequelae should be anticipated for retrolabyrinthine fracture lines that course into or through the VA. When considering treatment options in cases similar to the present, our findings suggest that surgical interventions targeting the eES (ES shunting/decompression procedures (31–33), which are used in MD with the intention to drain the hydropic endolymphatic fluid space, or to improve the fluid resorptive functions of the eES, respectively, most likely cannot work as proposed, because of the obliterated VA that separates the eES from the other labyrinthine fluid spaces.

Limitations of this case review concern events in the patient's medical history that may be confounding factors in the etiology of Meniere's symptoms: At age 27, the patient received chemotherapeutic treatment with cisplatin for a neoplastic disease (extragonadal germ cell tumor in the mediastinum). Although cisplatin can have cochleo-/vestibulotoxic side effects, PTA after chemotherapy showed no significant threshold changes compared to pretreatment thresholds. Moreover, no association between cisplatin treatment and Meniere's-like symptoms has been reported in the literature. The differential diagnosis of “delayed endolymphatic hydrops”—i.e., the onset of episodic vestibular symptoms years after a profound sensorineural hearing loss, e.g., of traumatic etiology, manifested in the ipsilateral (34, 35) or contralateral inner ear (36) and the postmortem histopathological finding of endolymphatic hydrops—must be considered in the present case. However, the hearing loss in the present case was fluctuating and overall slowly progressive, and no significant delay between the onset of fluctuating auditory and vestibular symptoms was documented, which renders the diagnosis of (ipsilateral) delayed endolymphatic hydrops unlikely in the present case. Finally, a coincidental idiopathic nature of Meniere's symptoms, by definition, cannot be excluded.

In conclusion, we hypothesize that posttraumatic Meniere's syndrome and MD, although of fundamentally different etiologies, presumably share a common pathophysiological basis, i.e., the functional loss of the eES epithelium. Radiological assessment of TB fractures should consider retrolabyrinthine fractures in the opercular region, which affect the VA but spare other labyrinth portions. Such fractures are rare but appear to cause distinctive clinical sequelae, i.e., delayed Meniere's syndrome.

## Data Availability

All datasets generated for this study are included in the manuscript and/or the supplementary files.

## Ethics Statement

Written informed consent for the publication of this case report was obtained from the patient.

## Author Contributions

AE conceived and designed the study. DB, MG, and AE performed 3D reconstructions and analyzed the imaging data. DB and AE prepared the figures and wrote the manuscript. BS, JN, JA, and AH provided critical review of the manuscript.

### Conflict of Interest Statement

The authors declare that the research was conducted in the absence of any commercial or financial relationships that could be construed as a potential conflict of interest.
